# SPECT versus Planar Scintigraphy as a Clinical Aid in Evaluation of the Elderly with Knee Pain

**DOI:** 10.1155/2013/842852

**Published:** 2013-01-22

**Authors:** Amir Oron, Izhar Arieli, Tamir Pritsch, Einat Even-Sapir, Nahum Halperin, Gabriel Agar

**Affiliations:** ^1^Department of Hand Surgery, Kaplan Medical Center, Hebrew University, P.O. Box 100, Rehovot, Israel; ^2^Department of Orthopedic Surgery, Kaplan Medical Center, Hebrew University, P.O. Box 100, Rehovot, Israel; ^3^Division of Orthopedics, Tel Aviv Sourasky Medical Center, 10 Weizmann Street, 64239 Tel Aviv, Israel; ^4^Imaging Division, Department of Nuclear Medicine, Tel Aviv Sourasky Medical Center, 10 Weizmann Street, 64239 Tel Aviv, Israel; ^5^Department of Orthopedics “A”, Assaf Harofeh Medical Center, 70300 Zerifin, Israel

## Abstract

Chronic knee pain is a common complaint among the elderly and appears in 30%–40% of the population over the age of 65. This study was performed in order to evaluate correlation between clinical presentation of chronic knee pain and the imaging findings of SPECT and planar bone scintigraphy. *Methods*. We prospectively recruited 116 patients over the age of 50 who had neither knee surgery nor trauma. Patients were divided into symptomatic and asymptomatic groups. All patients were examined by an experienced orthopedic surgeon; on the same day imaging was performed. Statistical analysis was performed to correlate physical examination findings with planar scintigraphy and SPECT findings and blood pool images. *Results*. In symptomatic patients, planar scintigraphy correlated significantly (*P* < 0.01) with the presence of excessive joint fluid, synovial condensation, and decrease in range of motion as measured in extension and flexion and patellar grinding test. SPECT findings correlated with all of the above tests as well as with medial and patellofemoral joint tenderness. *Conclusions*. We believe a finding of tenderness at the medial articular crease or of the patellofemoral compartment of the knee should be considered an indication for the use of SPECT scintigraphy rather than planar scintigraphy.

## 1. Introduction

Bone scans have become a key tool in assessing musculoskeletal pathology [1, 2]. Use of Single Photon Emission Computerized Tomography (SPECT) is becoming more common over the years as well [3]. Use of SPECT allows a three-dimensional assessment of the isotope dispersed in the subjects body, as compared with a two-dimensional assessment with planar “regular” scintigraphy. Bone scintigraphy is divided into three consecutive phases. The Perfusion phase, assessed at 30 to 60 seconds following injection, the Blood-Pool phase assessed at 2 to 5 minutes following injection, and the late phase at 2 to 5 hours following injection [4]. The use of Polyphosphate compounds with the isotope Tc^99m^ and mainly Tc-MDP allows differentiating between pathologies within soft tissues surrounding the bone and those within the bone in the perfusion phase and that found in the late phase which is attributed to the chemical reaction of the polyphosphate compounds and the hydroxyappetite crystals within bone [5]. Planar scintigraphy of complex or large structures within the skeletal system does not relay an accurate three-dimensional anatomic image, while use of SPECT mapping allows for spatial localization of the pathology in the mapped organ [3].

The differential diagnosis of knee pain in the elderly population is wide [6]. The most common etiologies are osteoarthritis, osteonecrosis, and degenerative tears of the menisci [6]. Knee pain may be referred from the hip or lumbar spine, and vascular malformations of the lower limbs have been attributed to knee pain as well [7]. Use of diagnostic aids allows for improved anatomical localization of pathology in the knee [6]. 

Osteoarthritis is the most common joint disease and afflicts the knees of 30% to 40% of those over 65 years of age suffering from it. Local degenerative changes in weight bearing joints appear from the second decade of life. By the age of 40, degenerative processes within weight bearing joints may be detected in up to 90% of the population, although these are generally asymptomatic [8]. In 1994, the World Health Organization and the American Academy of Orthopedic Surgeons defined osteoarthritis as “the result of both mechanical and biologic events that destabilize the normal coupling of degradation and synthesis of articular cartilage and subchondral bone” [9]. The subchondral bone has an important role in the development of chondral pathology. In 1986, Radin and Rose suggested that the cartilage within the joint is susceptible to shearing forces at points of decline or rise in subchondral bone density and cartilage fibrilation appears at these sights. Recurrent microtrauma, even in a healthy joint, brings about reorganization of the subchondral bone and thus transitional areas of changing subchondral bone density  [10].

An important tool in assessment of knee osteoarthritis is planar scintigraphy performed following instillation of marked Technitium [11]. In these patients an enhanced uptake in subarticular bone during the late phase may precede the roentgenogrphic changes by many years and relays the changes in local vascularity and the osteoblastic actitivity which mark the initial stages of osteoarthritis [11].

A good correlation was found between pathological roentgenograms and bone scintigraphy in osteoarthritic patients and these changes may be predicted by scintigraphic findings [12–14]. A diffuse uptake was correlated with pain and osteophyte formation, whereas localized uptake was correlated to subchondral bone sclerosis. A negative planar bone scintigraphy was found to be a good prognostic sign [15]. 

2-to-5 minutes following instillation of the isotope containing fluid to the circulation the blood pooling phase of the scan is elicited [4]. Assessment at this stage elicits inflammatory changes within the skeleton and soft tissues surrounding it [14, 16], due to concentrations of the isotope containing fluid in areas of hyperemia and neovascularization [17]. During periods rendered as “active osteoarthritis”, synovitis may be seen as an exaggerated pooling during the blood pool phase, which is positively correlated to osteoarthritic knee pain [15]. Collier and Johnson found SPECT to be superior to planar scintigraphy in patellofemoral osteoarthritis when concomitant involvement of one of the other knee compartments was evident as well, which was attributable to its ability to distinguish between the compartments which overlie each other in the planar view [12, 18, 19]. Both SPECT and planar scintigraphy were found inadequate in assessing the lateral compartment, although SPECT was superior to planar scintigraphy to some extent. As far as the medial compartment is concerned no advantage in the use of SPECT over planar scintigraphy was found in assessing osteoarthritis, although a pathological reading in the SPECT mapping may point to early subchondral changes in articular cartilage [18].

Aseptic necrosis of the medial femoral condyle is the corner stone of diagnosing osteonecrosis. The typical presenting symptom is pain along the medial articular aspect which is greater than that usually accompanying osteoarthritis [6]. Osteonecrosis typically afflicts the older population with predominance in women and may progress to osteoarthritic changes secondary to the disease. In the early stages of the disease, increased uptake is recorded in the initial blood flow phase and the late phase. The image of localized uptake in the medial femoral condyle in the SPECT mapping usually appears before that in planar scintigraphy [15, 18].

Meniscal pathology may cause knee pain as well, mainly in the medial compartment. The patient will describe a “locking” or “giving way” sensation of the knee. At times, excess joint fluids and localized tenderness over the articular crease may be appreciated as well [6]. In the younger patient population meniscal tears are usually traumatic in origin whereas in the elderly they are attributed to degenerative processes within the joint [6]. A number of clinical tests may be elicited in the knee with meniscal pathology with localized tenderness over the articular crease, and positive Mcmurry and Appley tests being the most widely accepted [20]. Fowler and Lubliner found the Mcmurry sign to be specific in meniscal tear associated with a limitation in knee extension [21], whereas others found limitations in the use of this test [20]. As to the Appley test, the literature attributes a low predictive value to this test in predicting meniscal pathology [20]. Localized tenderness over the articular crease is of high sensitivity and specificity in lateral meniscal tears whereas in medial meniscal tears these values are considerably lower. It should be pointed out that these findings are mainly from a relatively young patient population with a traumatic meniscal injury pattern, which may very well affect the clinical presentation in such cases [20]. Meniscal tears appear in SPECT mappings as localized uptake in a semicircular form in the vicinity of the suspected tibial plateau [3, 22, 23]. It has been found that degenerative “chronic” tears may mimic localized active osteoarthritis as well [18, 22].

Knee pain is a common complaint among the elderly. The orthopedist is often faced with the question which is the best diagnostic modality suitable for affirming his clinical diagnosis. Often, the roentgenographic appearance does not add to the confirmation of the suspected diagnosis since changes are late in most clinical pictures that is, osteoarthritic changes, avascular necrosis, and meniscal tears. In his effort to afford an objective finding to the elderly patients' knee pain, a growing number of diagnostic tests are performed, planar scintigraphy and SPECT becoming ever so popular. This study was performed in an effort to correlate findings in physical assessment of the knee with those in planar and SPECT scintigraphy, in an effort to better delineate the clinical value of each of these studies in the elderly with knee pain. 

## 2. Patients and Materials

116 patients were prospectively enrolled in this study. The patient population included 68 patients who complained of chronic knee pain and a control patient group which included 48 patients asymptomatic as to knee pain. Patient recruitment was performed at both Sourasky Medical Center in Tel-Aviv and at Assaf Harofeh Medical Center in Zerifin, Israel. All scans were performed at the Sourasky Medical Center Nuclear Medicine Department. The study was authorized by the local Helsinki committee. Following patient informed consent to enroll in the study, each patient was independently interviewed and physically examined. Inclusion criteria for enrollment in the study included: age over 50 years, patient willingness to undergo both planar and SPECT scintigraphy of both knees, agreement to undergo physical examination, fill in questionnaires and pain response scales, and patient health was considered “good” by the examining physician—that is, patient was mobile without use of assistance or devices and was able to take part in the study. Patients were excluded from the study if trauma involving the knees occurred in the previous two months, if pregnant or suspected to be so, patients with a known allergy to Technetium or its derivatives, and patients with history of Gout, Rheumatoid Arthritis, Psoriatic arthritis, or other known inflammatory disease. The patient incurred a fracture to one of his knees in the past or a surgical procedure involving either of his knees in the last six months. Patient participation in another clinical trial during participation within this trial was an exclusion criterion as well. 

Every participant filled out two questionnaires pertaining to knee pain. These were a Visual Analogue Pain Scale (VAS) and the Western Ontario and Mcmaster Universities Osteoarthritis Index (WOMAC). These are well accepted in the evaluation of knee pain [24]. Through interpretation of the patient responses to the questionnaires they were divided into a study (patient rendered symptomatic as to chronic knee pain) and control group.

All patients were evaluated by an experienced senior orthopedic surgeon (NH). The evaluation included the following measures; observation: angulation of the joint (varus versus valgus), the degree of freedom in rotation of the joint, and periarticular edema. Palpation: synovial fluid bulging, medial, lateral, patellofemoral, and popliteal sensitivity. Range of motion was assessed in flexion and extension. Stability of the joint and specific physical signs assessed included the Mcmurry test, Appley test, Compression & Traction test, Bounce home test, Anterior and Posterior drawer signs, and Medio-Lateral stability. Patellofemoral grinding test and *Q* angle were assessed as well.

All patients underwent planar and SPECT scintigraphy according to the following protocol. Following intravenous instillation of 25 mCi of Technetium MDP-99m, a dynamic assessment of five seconds per scan per minute was performed, and then a blood pool assessment was performed with collection of 500 K counts in an anterior view of the lower limbs. The planar scintigraphy was performed utilizing an anterior view with accumulation of 700 K counts. SPECT was performed up to a 180 degree angle with step angle of 3 degrees. Matrix was set as 64 × 64 and a zoom of 1.28 at a speed of eighteen seconds per step. Reconstruction was performed utilizing the Filtered Back Projection method with a 3.10 Mz filter, so a transaxial, saggittal, and coronal as well as a three-dimensional reconstruction was formed. Scintigraphies were assessed in order to relay local concentrations and there intensity with respect to knee anatomy. 

Both physical and radiological assessments were performed without the physicians performing them aware to which study group the patient belongs.

Statistical analysis was performed using the SAS statistical analysis system (SAS Institute Inc., Cary, North Carolina, USA). Analysis was performed on ninety-six knees (48 patients) in the control asymptomatic group of patients and on one hundred and thirty two knees in the symptomatic trial group (68 patients). Chi-square probability values were calculated utilizing the Frequency Procedure or in some cases the ANOVA procedure. For continuous variables such as range of motion *F*, and probability values were calculated utilizing the GLM Procedure or ANOVA analysis.

## 3. Results

Within the scope of this study we prospectively assessed 116 patients. Forty-eight patients were assigned to the control group and sixty-eight were assigned to the study-symptomatic group. The average patient age of those assigned to the study group was 64.735 years with a standard deviation of 10.316 and that of the control group was 66.354 with a standard deviation of 10.771. The study-symptomatic group of patients was comprised of 42 women (62%) and 26 men (38%) while the control-asymptomatic group was comprised of 17 women (35%) and 31 men (65%). The gender differences between the groups were statistically significant (*P* < 0.001)—percentage of women within the study group was 62% compared to a mere 35% in the control group. 

Patients enrolled in the study were divided into a study “symptomatic” group as to knee pain and a control “asymptomatic” group based on their responses on the VAS and WOMAC questionnaires. The average VAS response in the study group was 77.15 with a standard deviation of 42.7 and that of the control group was 1.23 with a standard deviation of 3.4 which was found to be statistically significant (*P* < 0.001). The average WOMAC response in the study group was 745.26 with a standard deviation of 634.9 and that of the control group was 13.96 with a standard deviation of 29.7 which was found to be statistically significant (*P* < 0.001) as well.

Planar and SPECT scintigraphy results were assessed according to the localization and intensity of the mapping in the respective compartments of the knee. The lateral compartment was divided into areas rendered as the Lateral Femoral Condyle and Lateral Tibial Plateau. The medial compartment was divided into areas rendered as the Medial Femoral Condyle and Medial Tibial Plateau. The patellofemoral compartment was divided into areas rendered Patellomedial and Patellolateral, whilst in the SPECT scans an area rendered Retropatellar was assigned. When the Blood Pooling phase was assessed, the scans were evaluated according to their uptake patterns rather than according to the different knee compartments.

The following tables contain data relevant to correlations between the different mapping methods and findings during the patients' physical examination. 

Incorporated in these tables are abbreviations which represent the following.

BLPAT: the uptake pattern in the Blood Pool phase. BLPOW: the relative amount of uptake in the Blood Pool phase. LatFmPs: the uptake pattern at the Lateral Femoral Condyle. LatFmPw: the power of local uptake at the Lateral Femoral Condyle. LatTiPs: the uptake pattern at the Lateral Tibial Plateau. LatTiPw: the power of uptake at the Lateral Tibial Plateau. PtMedPs: the uptake pattern at the Medial Patellar aspect. PtMedPw: the power of uptake at the Medial Patellar Aspect. PtLatPs: the uptake pattern at the Lateral Patellar aspect. PtLatPw: the power of uptake at the Lateral Patellar Aspect. RetPtPs: the uptake pattern at the Retro-Patellar aspect. RetPtPw: the power of uptake at the Retro-Patellar aspect. Alignment: knee alignment (Varus or Valgus). Rotation: limitation in knee rotation. Fluid: a finding of excessive fluid in the knee joint. Synovia: synovial fluid inflamation. MEDJO: Medial joint crease tenderness. LATJO: Lateral joint crease tenderness. PATJO: Patellar joint tenderness. POPFOS: Tenderness to palpation of the Popliteal Fossa. BAKER: tenderness to palpation of Baker's cyst. PES: tenderness at the Pes Anserinus area. BHT: Bounce home test. EXT: Extension range of the knee. FLEX: Flexion range of the knee. Mcmurry: Mcmurry meniscal test. Appley: Appley test for meniscal pathology. COMPRES: Compression & Traction test. ANTDRW: Anterior Drawer test. POSTDRW: Posterior Drawer test. MEDSTA: medial stability. LATSTA: lateral stability. PATGRIN: Patellar Grinding test. QANGLE: *Q* angle value. 

The values in bold are those with statistically significant Chi-square probability values. In order to prevent skewing of the radiological findings' interpretation it was decided that a correlation rendered relevant to statistical analysis would be between positive findings on physical examination and in both localization and amount of uptake in the examined scan.

A correlation between physical assessment findings and the results during the blood pool phase was found only in the study group. Such a correlation was found for physical findings such as excessive joint fluid, synovial inflammation, patellofemoral tenderness, and a restricted range of motion in both flexion and extension. It should be noted that in the control group probability values were not calculated for the joint Rotation examination, Bounce home test, Mcmurry and Appley meniscal tests, the Compression & Traction test, the Posterior Drawer test, and Lateral stability tests. This was due to the fact that no positive tests were found within the control group of patients. Due to the same reason, Chi-square probability for the joint Rotation test and Posterior Drawer sign were not calculated in the study group.

The following tables present Chi-square probability for physical assessment findings correlated with findings on both planar scintigraphy and SPECT, within the different knee compartments and study populations.

In Tables  2, 3, 4, 5, 6, and 7 the bolded values are Chi-square probability values of statistical significance (*P* < 0.01). Here too, in order to prevent skewing of the radiological findings' interpretation it was decided that a correlation rendered relevant to statistical analysis would be between positive findings on physical examination and in both localization and amount of uptake in the examined scan.

In the study-symptomatic patient group we found physical findings of excessive joint fluid, synovial thickening, limited joint range of motion, and positive patellar grinding tests to be associated with pathological findings in both planar scintigraphy and SPECT. When compared with Table 1, it is notable that all these physical findings but a positive patellar grinding test were correlated with excessive uptake during the blood pool phase. It should be noted that when the symptomatic patients were evaluated using SPECT a significant correlation was found between excessive tenderness at the medial joint crease and patellofemoral compartment on physical examination with positive findings on the SPECT examination. Such a correlation was not found when assessing the planar scintigraphy findings. 

A number of additional differences were found between the assessments of patients using the SPECT compared with planar scintigraphy. While planar scintigraphy findings showed a correlation between findings of excessive joint fluid and synovial proliferation with excessive uptake on planar scintigraphy at both the Medial Femoral Condyle and the Medial Tibial Plateau, these findings correlated with excessive uptake only at the Medial Tibial Plateau when SPECT was used. It was also found that a restricted range of knee flexion correlated well with a finding of excessive uptake at the Medial Femoral Condyle on planar scintigraphy whereas SPECT correlated this finding with excessive retropatellar uptake. Limited extension of the knee correlated with excessive uptake at the lateral and medial femoral condyles and the Medial Tibial Plateau in both planar scintigraphy and SPECT. 

A positive patellar grinding test correlated with excessive uptake at the Medial Tibial Plateau on planar scintigraphy and at both the Medial Tibial Plateau and the Lateral Femoral Condyle when evaluated using SPECT. 

A number of physical findings did not correlate with either excessive uptake on planar scintigraphy or in the use of SPECT. These findings were knee alignment, *Q* angle, lateral joint crease tenderness, tenderness over Baker's cyst, or in the vicinity of the Pes Anserinus, meniscal tests and tests for knee joint instability. It should be noted that for knee joint rotation and posterior drawer sign Chi-square probability values were not calculated, due to the absence of participants with pathological findings. 

In the control-asymptomatic patient group a number of physical findings correlated with planar scintigraphy. These included excessive medial joint crease tenderness with excessive uptake at the lateral femoral condyle, patellofemoral tenderness and excessive uptake at the Lateral Femoral Condyle and tenderness at the vicinity of the Pes Anserinus, and uptake at the Medial Tibial Plateau. 

When evaluated by SPECT, a correlation was found between tenderness to palpation of the lateral joint crease or at the patellofemoral compartment with excessive uptake at the Lateral Femoral Condyle was noted. A positive Appley test correlated with excessive uptake at the Medial Femoral Condyle. 

## 4. Discussion

Previously published studies stated that SPECT scintigraphy is superior to planar scintigraphy when osteoarthritis of the patellofemoral compartment is present concomitantly with osteoarthritis in the medial or lateral compartments of the knee [1, 16, 17]. It was also stated in previous studies that SPECT has no advantage over that of planar scintigraphy in assessing the medial compartment of the knee [16]. Our findings show an advantage of use of SPECT over planar scintigraphy in delineation of pathology in both the medial and lateral compartments of the knee. 

We did not find a correlation between localized lateral compartment sensitivity in neither planar nor SPECT scintigraphy. These findings do align with those of a previous study by Yang et al. which found both methods to be of poor clinical value in evaluation of the lateral compartment of the knee [16]. 

When reviewing the results the physical findings of intraarticular fluid, synovial proliferation, limitations in range of motion, and the positive patellar grinding test were found to correlate positively with both planar and SPECT scintigraphy as well as a pathological reading during the blood pool phase. Previous studies have found pathological readings during the blood pool phase to be correlated with an inflammatory condition in the localized area of the skeletal system. Thus, objective findings in scintigraphy which were correlated with the above physical findings further corroborate our assumption that within the symptomatic patient population the knee pain stems from osteoarthritic changes. This may be further strengthened when reemphasizing that patients enrolled in the study did not suffer from trauma or undergo surgical intervention prior to enrollment in the study. 

SPECT was superior to planar scintigraphy in correlating localized medial articular crease and patellofemoral tenserness with uptake in the medial tibial plateau. Further advantages incurred through use of the SPECT mapping were probably due to its ability to better define anatomic areas and delineate localized uptake. For example, synovial proliferation with a finding of excess intra-articular fluid were found to correlate positively to excess uptake at both the Medial Femoral and Tibial Condyles on planar scintigraphy whereas SPECT findings were limited to the tibia alone. A similar scenario, but of greater clinical impact was found when correlating limited flexion of the knee with planar scintigraphy findings which resulted in excessive uptake in the vicinity of the Medial Femoral Condyle while SPECT correlated with excessive uptake at the patellofemoral joint that is, where pathological findings would be expected in patients suffering from osteoarthritic changes and a flexion contracture.

Of interest is the fact that in the control group, which contained patients rendered asymptomatic as to knee pain, a positive correlation was found between tenderness at the lateral articular crease and patellofemoral compartment with excessive uptake in the lateral femoral condyle (Tables 8 and 9). This may be explained by changes in subchondral bone which may appear prior to articular chondral damage and thus  does not  eliminate knee pain in these patients. A positive Appley test correlated positively with excessive uptake in the medial femoral condyle in SPECT scintigraphy, as well. We could not find an acceptable explanation to this finding, whilst moreover other meniscal tests such as the Bounce home and Mcmurry tests failed to show any significant correlation whatsoever. 

It is of note that a positive correlation was found in planar scintigraphy between localized tenderness at the patellofemoral junction and excess uptake at the lateral femoral condyle which may be explained by localized overlapping and inferior capability of planar scintigraphy to differentiate uptake  emanating from the patellofemoral compartment from that of a true lateral articular joint nature.

This study was aimed at comparing planar and SPECT scintigraphy while correlating them to physical assessment findings in patients suffering from chronic knee pain and those asymptomatic as to knee pain. One of the difficulties encountered within the scope of this study was the absence of a clinically proven entity for knee pain in the symptomatic patient group. This could be coped with by performing a concomitant assessment of patients by knee arthroscopy which was not done within the scope of this study. Our working diagnosis in most, if not all, symptomatic patients assessed within the scope of this study, was that they suffer from osteoarthritis of the knee as indicated by their complaints and physical assessment. It should be noted that the symptomatic and asymptomatic patient populations differed significantly (*P* < 0.01) as to gender and were not of equal size, facts which may very well bring about a statistical sway in the study results.

Our findings suggest SPECT scintigraphy to be superior to planar scintigraphy when correlated to physical assessment findings in elderly patients suffering from chronic knee pain. When tenderness localized to the medial articular crease or the patellofemoral compartment was found during physical assessment these findings correlated positively with SPECT scintigraphy, whereas planar scintigraphy findings lacked any correlation. In an age when unicondylar knee arthroplasty is readily available as a treatment for unicompartmental osteoarthritis of the medial or lateral compartments of the knee [25, 26], an advantage exists in the ability to delineate sequestered compartmental involvement in the human knee. We believe a finding of tenderness at the medial articular crease or of the patellofemoral compartment of the knee should be considered an indication for the use of SPECT scintigraphy rather than planar scintigraphy as a diagnostic aid in assessing the elderly with chronic knee pain. 

## Figures and Tables

**Table 1:** Table 1 shows Chi-square probability and *F* values for the physical assessment findings and the Blood Pool phase in both the study and control groups.

	Control group		Study group	
	BLPAT	BLPOW	BLPAT	BLPOW
ALIGNMENT	0.7572	0.7747	0.1634	*0.0135 *
ROTATION	—	—	—	—
FLUID	0.7798	0.7891	**0.001**	**0.0028**
SYNOVIA	0.7798	0.7891	**0.010**	**0.0004**
MEDJO	0.3759	0.6537	0.4634	0.5211
LATJO	0.7527	0.7281	0.1869	0.3576
PATJO	0.2539	0.2346	**0.0043**	**0.0085**
POPFOS	0.0342	0.7090	0.2026	*0.0403 *
BAKER	0.7825	0.7917	*0.0156 *	0.0985
PES	0.5898	0.4891	0.5316	0.9231
BHT	—	—	0.1849	0.4358
EXT	*F*(2,93) = 2.03	*F*(2,93) = 2.05	**F**(3,131) = 4.73	**F**(3,131) = 9.63
	*P* = 0.1368	*P* = 0.1341	**P** = 0.0036	**P** < 0.0001
FLEX	*F*(2,93) = 2.55	*F*(2,93) = 1.73	**F**(3,131) = 5.10	**F**(3,131) = 6.60
	*P* = 0.0839	*P* = 0.1834	**P** = 0.0023	**P** = 0.0003
MCMURRY	—	—	0.2133	0.0662
APLEY	—	—	0.5838	0.1996
COMPRES	—	—	0.6686	0.8301
ANTDRW	0.7798	0.7891	0.7625	0.7438
POSTDRW	—	—	—	—
MEDSTA	0.6049	0.6194	0.5017	0.1523
LATSTA	—	—	0.3773	0.0941
PATGRIN	0.7716	0.6619	**0.0027**	*0.0289 *
QANGLE	0.7263	0.7020	0.7595	0.2462

**Table 2:** Table 2 shows the Chi-square probability for findings on physical assessment correlated with planar scintigraphy in the lateral compartment of the knee in both study groups.

	Control group				Study group			
	LatFmPs	LatFmPw	LatTiPs	LatTiPw	LatFmPs	LatFmPw	LatTiPs	LatTiPw
ALIGNMENT	0.2990	0.5249	0.9352	0.6368	0.2072	0.1746	*0.0454 *	0.1599
ROTATION	—	—	—	—	—	—	—	—
FLUID	0.7040	0.9174	0.9304	0.9304	0.1974	0.4380	0.8917	0.4412
SYNOVIA	0.7040	0.9174	0.9304	0.9304	0.0657	0.2469	0.1110	0.3345
MEDJO	**0.0049**	**<0.0001**	0.3149	0.0724	0.0423	0.4733	0.3453	0.3470
LATJO	0.0623	**<0.0001**	0.9985	0.9985	0.6371	0.7748	0.7725	0.9457
PATJO	**0.0078**	**<0.0001**	0.9304	0.9304	*0.0500 *	*0.0368 *	0.9752	0.9130
POPFOS	0.5891	0.8400	0.8642	0.8642	0.8241	0.9532	0.6107	0.4850
BAKER	0.7023	0.9164	0.9295	0.9295	*0.0164 *	0.1138	*0.0253 *	**0.0041**
PES	0.8112	0.9845	0.9872	0.9872	*0.0324 *	0.2790	0.5262	0.6993
BHT	—	—	—	—	0.0924	*0.0333 *	0.4261	0.1129
EXT	0.4193	0.6785	0.1041	*0.0112 *	**0.0003**	**<0.0001**	*0.0105 *	**0.0083**
FLEX	0.8625	0.8155	0.6502	0.8719	0.1142	0.0989	0.7759	0.7512
MCMURRY	—	—	—	—	0.7124	0.5590	0.7386	0.8772
APLEY	—	—	—	—	0.9040	0.8709	0.7595	0.8819
COMPRES	—	—	—	—	0.8045	0.9329	0.8134	0.9375
ANTDRW	0.7040	0.9174	0.9304	0.9304	0.8045	0.9329	0.8134	0.9375
POSTDRW	—	—	—	—	—	—	—	—
MEDSTA	0.5891	0.8400	0.8642	0.8642	0.6430	0.8295	0.2179	0.2116
LATSTA	—	—	—	—	0.7199	0.8832	0.7318	0.8908
PATGRIN	0.1110	0.0320	0.6331	0.6331	*0.0409 *	**0.0061**	0.3625	0.3731
QANGLE	0.4400	0.7003	0.7422	0.7442	0.5734	0.7741	0.5896	0.7876

**Table 3:** Table 3 shows the Chi-square probability for findings on physical assessment correlated with planar scintigraphy in the medial compartment of the knee in both study groups.

	Control group				Study group			
	MedFmPs	MedFmPw	MedTiPs	MedTiPw	MedFmPs	MedFmPw	MedTiPs	MedTiPw
ALIGNMENT	0.8207	0.7046	0.8352	0.7082	0.2267	0.1692	*0.2569 *	*0.0455 *
ROTATION	—	—	—	—	—	—	—	—
FLUID	0.5617	0.8450	0.7675	0.7580	**0.0068**	**0.0098**	**0.0010**	**<0.0001**
SYNOVIA	0.5617	0.8450	0.7675	0.7580	**0.0051**	**0.0001**	**0.0001**	**0.0007**
MEDJO	*0.9169 *	*0.9936 *	*0.0346 *	0.8127	0.2809	*0.0380 *	0.0701	*0.0149 *
LATJO	0.7934	*0.9844 *	0.9510	0.9453	0.4444	0.4532	0.1826	0.0590
PATJO	*0.5617 *	*0.8450 *	0.7675	0.7580	*0.0656 *	*0.1528 *	0.2800	*0.0343 *
POPFOS	0.4093	0.5438	0.8082	0.7285	0.0616	*0.0145 *	0.5306	0.5613
BAKER	0.0838	0.1325	0.3166	0.2616	*0.0462 *	**<0.0001**	*0.5861 *	*0.0848 *
PES	0.8302	0.9763	**0.0001**	**0.0096**	*0.0541 *	0.8275	0.0978	0.2501
BHT	—	—	—	—	**0.0055**	*0.0035 *	0.1471	0.3207
EXT	0.2163	0.4675	0.3010	0.8128	**0.0007**	**<0.0001**	**0.0027**	**<0.0001**
FLEX	0.9297	**0.0041**	0.3731	0.3129	**0.0029**	**0.0002**	0.1090	*0.0138 *
MCMURRY	—	—	—	—	0.1783	0.0897	0.9597	0.3634
APLEY	—	—	—	—	0.1176	*0.0399 *	0.2873	0.1656
COMPRES	—	—	—	—	0.1032	0.1248	0.0867	**0.0037**
ANTDRW	0.0817	0.1290	0.3112	0.2566	0.1032	*0.0497 *	0.9041	0.8771
POSTDRW	—	—	—	—	—	—	—	—
MEDSTA	0.4903	0.7115	0.0945	0.0640	0.8977	0.8977	0.5161	0.6965
LATSTA	—	—	—	—	0.4566	0.6784	0.1872	0.3677
PATGRIN	0.1441	0.3442	**0.0006**	0.6723	**0.0009**	*0.0151 *	**0.0033**	**0.0070**
QANGLE	1.000	0.8717	0.6471	0.4641	0.8512	0.6513	0.6523	0.0564

**Table 4:** Table 4 shows the Chi-square probability for findings on physical assessment correlated with planar scintigraphy in the patellofemoral compartment of the knee in both study groups.

	Control group				Study group			
	PtMedPs	PtMedPw	PtLatPs	PtLatPw	PtMedPs	PtMedPw	PtLatPs	PtLatPw
ALIGNMENT	0.6582	0.9926	0.6181	0.2916	0.9764	0.8887	*0.2033 *	0.2027
ROTATION	—	—	—	—	—	—	—	—
FLUID	0.3640	0.2566	0.3467	0.1828	0.8594	0.0964	0.1934	*0.0182 *
SYNOVIA	0.3640	0.2566	0.3467	0.1828	0.1060	**0.0043**	0.1570	**0.0010**
MEDJO	*0.3514 *	*0.0333 *	*0.0157 *	**0.0003**	0.2604	0.9584	0.1476	0.1487
LATJO	0.4019	*0.2144 *	0.5486	0.2754	0.6691	0.7969	0.2224	0.5221
PATJO	*0.3640 *	*0.2566 *	0.6970	0.7077	*0.0247 *	*0.7019 *	**0.0080**	0.9187
POPFOS	0.8333	0.6927	0.8130	0.5928	0.2854	0.7467	0.1024	0.3177
BAKER	0.3523	0.2430	0.3523	0.1870	*0.6375 *	*0.0241 *	*0.1226 *	**0.0002**
PES	0.1197	*0.0311 *	0.4173	0.1143	*0.2258 *	0.8044	0.4929	0.1024
BHT	—	—	—	—	0.0815	*0.3502 *	0.0667	0.1553
EXT	0.7777	0.6831	0.7469	0.7971	0.9091	0.1737	0.1673	**0.0001**
FLEX	0.5357	0.9808	0.1309	0.2241	0.6097	0.1050	0.6626	*0.0104 *
MCMURRY	—	—	—	—	*0.0333 *	0.1008	0.6740	0.7483
APLEY	—	—	—	—	0.1856	0.5619	0.7142	0.9866
COMPRES	—	—	—	—	0.0502	0.9231	0.0546	0.9352
ANTDRW	0.6970	0.6862	0.6970	0.7077	0.3192	0.3791	0.8969	0.9352
POSTDRW	—	—	—	—	—	—	—	—
MEDSTA	0.4822	0.4671	0.4822	0.4972	0.0855	0.1172	0.1689	0.2241
LATSTA	—	—	—	—	0.1603	0.2230	0.8407	0.8761
PATGRIN	*0.0268 *	**0.0057**	0.1630	*0.0272 *	*0.1847 *	*0.3415 *	0.0884	0.1043
QANGLE	0.6889	0.8693	0.6550	0.6317	0.7401	0.9925	0.5622	0.9578

**Table 5:** Table 5 shows the Chi-square probability values for findings on physical assessment correlated with SPECT in the lateral compartment of the knee in both study groups.

	Control group				Study group			
	LatFmPs	LatFmPw	LatTiPs	LatTiPw	LatFmPs	LatFmPw	LatTiPs	LatTiPw
ALIGNMENT	0.9458	0.7006	0.1673	0.6587	*0.0284 *	0.1184	*0.0246 *	*0.1633 *
ROTATION	—	—	—	—	—	—	—	—
FLUID	0.3290	0.2012	0.8370	0.9492	0.5651	0.7916	0.8864	0.6509
SYNOVIA	0.3290	0.2012	0.8370	0.9492	0.1680	0.1396	0.3715	0.8979
MEDJO	**<0.0001**	*0.0529 *	0.2444	0.4964	0.7767	0.1971	0.3737	**0.0076**
LATJO	**<0.0001**	**0.0008**	0.9820	0.9992	0.5536	0.8991	0.4677	0.5286
PATJO	**<0.0001**	**0.0039**	0.8370	0.9492	**0.0002**	*0.0162 *	0.0985	0.0549
POPFOS	0.1057	*0.0391 *	0.3837	0.5900	*0.0105 *	0.1530	0.4974	**0.0016**
BAKER	0.3166	0.1870	0.0675	0.1454	*0.0484 *	**0.0002**	*0.1436 *	*0.2500 *
PES	0.5437	0.3233	0.8149	0.9740	*0.5390 *	**0.0076**	0.0923	0.7481
BHT	—	—	—	—	0.2387	*0.3840 *	0.2225	0.3145
EXT	0.9672	0.8075	0.0833	0.1474	**0.0066**	**<0.0001**	0.1306	*0.0368 *
FLEX	0.3944	0.6383	0.0752	0.6883	0.2987	**0.0061**	0.8212	0.9441
MCMURRY	—	—	—	—	0.5113	0.6272	*0.0414 *	*0.0106 *
APLEY	—	—	—	—	0.5961	0.7880	*0.0380 *	**0.0078**
COMPRES	—	—	—	—	0.4200	0.8848	0.7318	0.8966
ANTDRW	0.7859	0.7859	0.8370	0.9492	0.3924	0.7304	0.7318	0.8966
POSTDRW	—	—	—	—	—	—	—	—
MEDSTA	0.6144	0.6144	0.6980	0.8687	0.9722	0.9625	*0.0375 *	**<0.0001**
LATSTA	—	—	—	—	0.0832	0.2110	0.4054	*0.0163 *
PATGRIN	**0.0005**	0.0729	0.6883	0.8621	**0.0032**	**0.0097**	0.2775	0.4863
QANGLE	0.3696	0.3696	0.4796	0.6893	0.8207	0.9634	0.5957	0.1036

**Table 6:** Table 6 shows the Chi-square probability values for findings on physical assessment correlated with SPECT in the medial compartment of the knee in both study groups.

	Control group				Study group			
	MedFmPs	MedFmPw	MedTiPs	MedTiPw	MedFmPs	MedFmPw	MedTiPs	MedTiPw
ALIGNMENT	0.1631	0.2182	0.5999	0.5061	0.1547	0.2091	*0.1320 *	**0.0012**
ROTATION	—	—	—	—	—	—	—	—
FLUID	0.5948	0.8605	0.7580	0.7768	*0.8517 *	*0.9540 *	**0.0060**	**0.0003**
SYNOVIA	0.5498	0.8605	0.7580	0.7768	*0.7471 *	*0.8934 *	**0.0099**	**0.0012**
MEDJO	*0.9016 *	*0.9162 *	*0.2380 *	0.2771	0.6892	*0.2763 *	**0.0014**	**0.0017**
LATJO	0.2469	*0.5510 *	0.7397	0.6971	0.9376	0.9642	0.9239	0.3330
PATJO	*0.5498 *	*0.8605 *	0.7580	0.7768	*0.0129 *	*0.0476 *	**<0.0001**	**0.0003**
POPFOS	0.3309	*0.0120 *	0.7792	0.0571	0.0706	**0.0007**	*0.0451 *	0.1218
BAKER	0.0516	0.0984	0.2984	**0.0017**	*0.8136 *	*0.8936 *	*0.2440 *	*0.0797 *
PES	0.8469	0.9470	*0.0979 *	*0.0277 *	*0.1222 *	0.2440	*0.0318 *	0.2220
BHT	—	—	—	—	*0.9412 *	*0.9130 *	0.0911	0.0989
EXT	0.2574	0.5078	0.0782	*0.8365 *	**0.0009**	**0.0025**	**0.0022**	**<0.0001**
FLEX	*0.0434 *	0.0823	0.8693	*0.9067 *	0.6287	0.6294	*0.0379 *	**0.0040**
MCMURRY	—	—	—	—	*0.0364 *	0.0842	0.5812	0.6365
APLEY	—	—	—	—	**0.0055**	**0.0091**	0.2376	0.0978
COMPRES	—	—	—	—	0.6747	0.8526	0.0088	*0.0614 *
ANTDRW	0.5948	0.8605	0.7580	0.7768	0.7440	*0.6974 *	0.7582	0.8374
POSTDRW	—	—	—	—	—	—	—	—
MEDSTA	0.4495	0.7381	**<0.0001**	0.0550	0.9661	0.5163	0.5191	0.5013
LATSTA	—	—	—	—	0.9455	0.3857	0.4009	0.7275
PATGRIN	0.7499	0.8547	*0.4577 *	0.5575	*0.0115 *	*0.0282 *	**0.0009**	**0.0019**
QANGLE	0.1645	0.2535	0.1366	0.3733	*0.0218 *	**0.0010**	0.2868	*0.0120 *

**Table 7:** Table 7 shows the Chi-square probability values for findings on physical assessment correlated with SPECT in the patellofemoral compartment of the knee in both study groups.

	Control group				Study group			
	PtMedPs	PtMedPw	PtLatPs	PtLatPw	PtMedPs	PtMedPw	PtLatPs	PtLatPw
ALIGNMENT	*0.4867 *	*0.4781 *	*0.4985 *	*0.4586 *	*0.0494 *	*0.1587 *	*0.0792 *	*0.2204 *
ROTATION	*— *	*— *	*— *	*— *	*— *	*— *	*— *	*— *
FLUID	*0.3640 *	*0.1412 *	*0.4929 *	*0.6725 *	*0.2080 *	*0.4641 *	*0.8876 *	*0.5358 *
SYNOVIA	*0.3640 *	*0.1412 *	*0.4929 *	*0.6725 *	*0.1616 *	*0.1671 *	*0.5050 *	*0.8314 *
MEDJO	*0.7567 *	*0.9400 *	*0.0709 *	*0.3517 *	*0.2477 *	*0.2942 *	*0.2880 *	*0.8385 *
LATJO	*0.6851 *	*0.7665 *	*0.1500 *	*0.6485 *	*0.3595 *	*0.4196 *	*0.5102 *	*0.4376 *
PATJO	*0.6159 *	*0.7883 *	*0.0291 *	*0.3608 *	*0.2462 *	*0.2258 *	*0.5566 *	*0.8241 *
POPFOS	*0.1297 *	*0.1936 *	*0.2991 *	*0.6752 *	*0.0296 *	*0.0233 *	*0.7122 *	*0.4781 *
BAKER	*0.3698 *	*0.5125 *	*0.5567 *	*0.3671 *	*0.0812 *	*0.3539 *	*0.2523 *	*0.3792 *
PES	*0.1486 *	*0.3352 *	*0.1117 *	*0.1667 *	*0.1662 *	*0.1805 *	*0.9667 *	*0.8314 *
BHT	*— *	*— *	*— *	*— *	*0.6883 *	*0.4029 *	*0.4818 *	*0.5574 *
EXT	*0.4610 *	*0.8594 *	*0.6576 *	*0.5799 *	*0.3802 *	*0.5156 *	*0.1197 *	*0.1234 *
FLEX	*0.2906 *	*0.8298 *	*0.5738 *	*0.5866 *	*0.5024 *	*0.1068 *	*0.7451 *	*0.0406 *
MCMURRY	*— *	*— *	*— *	*— *	*0.9053 *	*0.1328 *	*0.7643 *	*0.9467 *
APLEY	*— *	*— *	*— *	*— *	*0.7680 *	*0.3041 *	*0.3185 *	*0.5135 *
COMPRES	*— *	*— *	*— *	*— *	*0.8001 *	*0.6545 *	*0.6907 *	*0.0843 *
ANTDRW	*0.3640 *	*0.5059 *	*0.4929 *	*0.6725 *	*0.8001 *	*0.8180 *	*0.3732 *	*0.3362 *
POSTDRW	*— *	*— *	*— *	*— *	*— *	*— *	*— *	*— *
MEDSTA	*0.3755 *	*0.5460 *	*0.2991 *	*0.2928 *	*0.6359 *	*0.5955 *	*0.1352 *	*0.3865 *
LATSTA	*— *	*— *	*— *	*— *	*0.6694 *	*0.7702 *	*0.2255 *	*0.6029 *
PATGRIN	*0.4322 *	*0.2826 *	**0.0071**	*0.1874 *	*0.5874 *	*0.7193 *	*0.8839 *	*0.9198 *
QANGLE	*0.6063 *	*0.2804 *	*0.3624 *	*0.7035 *	*0.0791 *	*0.0447 *	*0.8936 *	*0.7289 *

	RetPtPs	RetPtPw			RetPtPs	RetPtPw		

ALIGNMENT	*0.2691 *	*0.3501 *			*0.1409 *	*0.5555 *		
ROTATION	*— *	*— *			*— *	*— *		
FLUID	*0.6862 *	*0.8363 *			*0.0240 *	*0.0364 *		
SYNOVIA	*0.6862 *	*0.8363 *			*0.0326 *	*0.0884 *		
MEDJO	*0.5818 *	*0.6625 *			*0.5712 *	*0.7996 *		
LATJO	*0.5178 *	*0.4640 *			*0.4778 *	*0.8283 *		
PATJO	*0.6862 *	*0.8363 *			*0.0876 *	*0.2477 *		
POPFOS	*0.4671 *	*0.6307 *			*0.4353 *	*0.9277 *		
BAKER	*0.6814 *	*0.8322 *			*0.3530 *	*0.1527 *		
PES	*0.6940 *	*0.4988 *			*0.6045 *	*0.8145 *		
BHT	*— *	*— *			*0.0537 *	*0.3714 *		
EXT	*0.2504 *	*0.5800 *			*0.0197 *	**0.0015**		
FLEX	*0.5313 *	*0.9295 *			**0.0010**	**0.0020**		
MCMURRY	*— *	*— *			*0.0227 *	*0.1131 *		
APLEY	*— *	*— *			*0.2539 *	*0.4998 *		
COMPRES	*— *	*— *			*0.2475 *	*0.0578 *		
ANTDRW	*0.6862 *	*0.8363 *			*0.8480 *	*0.7694 *		
POSTDRW	*— *	*— *			*— *	*— *		
MEDSTA	*0.7855 *	*0.8348 *			*0.3851 *	*0.6382 *		
LATSTA	*— *	*— *			*0.6459 *	*0.6930 *		
PATGRIN	*0.4694 *	*0.2215 *			*0.0646 *	*0.1049 *		
QANGLE	*0.6842 *	*0.9563 *			*0.5754 *	*0.8115 *		

**Table 8:** Table 8 summarizes the statistically significant differences in Chi-square values of the relative knee compartments in each of the patient groups and mapping modalities.

	Scintigraphy		SPECT	
	Control	Study	Control	Study
ALIGNMENT	—	—	—	—
ROTATION	—	—	—	—
FLUID	—	MedFm, MedTi	—	MedTi
SYNOVIA	—	MedFm, MedTi	—	MedTi
MEDJO	LatFm	—	—	MedTi
LATJO	—	—	LatFm	—
PATJO	LatFm	—	LatFm	MedTi
POPFOS	—	—	—	—
BAKER	—	—	—	—
PES	MedTi	—	—	—
BHT	—	—	—	—
EXT	—	LatFm, MedFm, MedTi	—	LatFm, MedFm, MedTi
FLEX	—	MedFm	—	RetPt
MCMURRY	—	—	—	—
APLEY	—	—	MedFm	—
COMPRES	—	—	—	—
ANTDRW	—	—	—	—
POSTDRW	—	—	—	—
MEDSTA	—	—	—	—
LATSTA	—	—	—	—
PATGRIN	—	MedTi	—	LatFm, MedTi
QANGLE	—	—	—	—

**Table 9:** Table 9 summarizes the correlations found between excessive tenderness to palpation in the different knee compartments and planar scintigraphy and SPECT in the study symptomatic patient group.

Mapping method	Pattern and power of uptake in different knee compartments	Excessive tenderness to palpation at medial knee compartment	Excessive tenderness to palpation at lateral knee compartment	Excessive tenderness to palpation at patellofemoral knee compartment
Planar scintigrphy	LatFmPs	*0.0423 *	*0.6371 *	*0.0500 *
	LatFmPw	*0.4733 *	*0.7743 *	*0.0368 *
	LatTiPs	*0.3453 *	*0.7725 *	*0.9752 *
	LatTiPw	*0.3470 *	*0.9457 *	*0.9130 *
	MedFmPs	*0.2809 *	*0.4444 *	*0.0656 *
	MedFmPw	*0.0380 *	*0.4532 *	*0.1528 *
	MedTiPs	*0.0701 *	*0.1826 *	*0.2800 *
	MedTiPw	*0.0149 *	*0.0590 *	*0.0343 *
	PtMedPs	*0.2604 *	*0.6691 *	*0.0247 *
	PtMedPw	*0.9584 *	*0.7969 *	*0.7019 *
	PtLatPs	0.1476	0.2224	**0.0080**
	PtLatPw	*0.1487 *	*0.5221 *	*0.9187 *

SPECT	LatFmPs	0.7767	0.5536	**0.0002**
	LatFmPw	*0.1971 *	*0.8991 *	*0.0162 *
	LatTiPs	*0.3737 *	*0.4677 *	*0.0985 *
	LatTiPw	**0.0076**	*0.5286 *	*0.0549 *
	MedFmPs	*0.6892 *	*0.9376 *	*0.0129 *
	MedFmPw	*0.2763 *	*0.9642 *	*0.0476 *
	MedTiPs	**0.0014**	0.9239	**<0.0001**
	MedTiPw	**0.0017**	0.3330	**0.0003**
	PtMedPs	*0.2477 *	*0.3595 *	*0.2462 *
	PtMedPw	*0.2942 *	*0.4196 *	*0.2258 *
	PtLatPs	*0.2880 *	*0.5102 *	*0.5566 *
	PtLatPw	*0.8385 *	*0.4376 *	*0.8241 *
	RetPtPs	*0.5712 *	*0.4778 *	*0.0876 *
	RetPtPw	*0.7996 *	*0.8283 *	*0.2477 *
